# Characteristics of circular RNA expression of pulmonary macrophages in mice with sepsis‐induced acute lung injury

**DOI:** 10.1111/jcmm.14577

**Published:** 2019-08-14

**Authors:** Xiaowei Bao, Qianqian Zhang, Na Liu, Shougang Zhuang, Zhe Li, Qingshu Meng, Hong Sun, Jianwen Bai, Xiaohui Zhou, Lunxian Tang

**Affiliations:** ^1^ Department of Internal Emergency Medicine and Critical Care, Shanghai East Hospital Tong Ji University Shanghai China; ^2^ Medical School/Tongji University Shanghai China; ^3^ Department of Nephrology, Shanghai East Hospital Tongji University School of Medicine Shanghai China; ^4^ Department of Medicine, Rhode Island Hospital and Alpert Medical School Brown University Providence RI USA; ^5^ Research Center for Translational Medicine, Shanghai East Hospital Tongji University Shanghai China

**Keywords:** acute lung injury, CircRNAs, macrophage polarization, miRNA, sepsis

## Abstract

Circular RNAs (circRNAs) make up a large class of non‐coding RNAs and play important roles in the pathology of a variety of diseases. However, their roles in pulmonary macrophage polarization after sepsisinduced lung injury is unknown. In this study, mice were divided into two groups: Sham control group and cecal ligation and puncture (CLP)‐induced ALI group. Macrophages were isolated from lung homogenates 24 hours after SCLP/CLP. We started with RNA‐seq of circRNA changes in macrophages and validated by RT‐PCR in the following experiments. A total of 4318 circRNAs were detected in the two groups. Of these, 11 and 126 circRNAs were found to be significantly upregulated and downregulated, respectively, compared to the control (p≤0.05, Fold Change ≥2). Differentially expressed circRNAs with a high foldchange (fold‐change >4, P<0.05) were selected for validation by qRT‐PCR, 10 of which were verified. Furthermore, the most differentially expressed circRNAs within all the comparisons were annotated in detail with circRNA/miRNA interaction information using miRNA target prediction software. The network of circRNA‐miRNA‐mRNA was illustrated by cytoscape software. Gene ontology analyses indicated the upregulated circRNAs were involved in the multiple biological functions such as regulation of mitochondrion distribution and Notch binding, while the down‐regulated circRNAs mainly involved in the biological process as histone H3K27 methylation. KEGG pathway analysis revealed TGF‐beta signaling pathway was related to the upregulated circRNAs. The present study provides a novel insight into the roles of circRNAs in pulmonary macrophage differentiation and polarization post septic lung injury.

## 1 INTRODUCTION

Acute lung injury (ALI)/acute respiratory distress syndrome (ARDS) remains a devastating disease associated with high mortality in critically ill patients.^1^ Currently, there is increasing evidence suggesting that macrophages are key factors in the pathogenesis of ALI/ARDS.^2^ Circular RNAs (circRNAs) are a class of newly identified non‐coding RNAs and play crucial roles in regulating gene expression, functioning as miRNA “sponge” to suppress miRNA activity,^3^ regulating transcription of genes, and translating protein genes.^3^ However, the role of circRNAs in gene expression occurring during macrophage polarization is still relatively rare. Only recently, a report had documented the general changes of circRNAs in two distinct polarizing conditions (M_1_ and M_2_ macrophages) in vitro.^4^ To date, no report was made on the expression profiles of circRNAs during macrophage activation after cecal ligation and puncture (CLP)‐induced ALI/ARDS.

## 2 MATERIALS AND METHODS

### 2.1 Mouse model of CLP‐induced ALI

All animal experiments were performed according to the guidelines for the Care and Use of Laboratory Animals (Ministry of Health, China, 1998). Experiments were conducted under protocols approved by the Animal Use Committee of East Hospital/Tongji University. A mice model of CLP‐induced ALI was created as previously reported.^5^, ^6^

### 2.2 Pulmonary macrophage isolation and RNA isolation

The mice were divided into sham control group and CLP group (N = 4 for each group). All animals were sacrificed 24 hours after SCLP/CLP insult, and the F4/80^+^ pulmonary macrophages were isolated as previously reported.^5^, ^6^ RNA was isolated using TRIzol reagent (Ambion) according to the manufacturer's instructions.

### 2.3 High‐throughput sequencing and analysis of circRNA

High‐throughput whole transcriptome sequencing and subsequent bioinformatics analysis were performed by Cloud‐Seq Biotech as previously reported .^7^

### 2.4 qRT‐PCR validation of circRNAs

Quantitative real‐time PCR (qRT‐PCR) was used to validate circRNA expression as previously reported.^5^, ^6^, ^7^ Of the circRNAs identified, three up‐regulated and seven down‐regulated circRNAs were selected for validation.

### 2.5 Gene ontology and KEGG pathway analysis of selected circRNAs

Gene ontology (GO) and KEGG analyses were performed for differentially expressed circRNA‐associated genes. The top 10 enriched GO terms among the two groups were highlighted as differentially expressed. KEGG pathway analysis was performed to identify the pathways associated with circRNA‐targeted miRNAs.

### 2.6 Prediction of circRNA‐miRNA‐mRNA interactions

CircRNA‐miRNA interaction was predicted by popular target prediction softwares, and network was constructed by Cytoscape software. For each circRNA, we showed the top 5 miRNAs that potentially bind to the circRNA and the five most likely target genes to every miRNAs.

### 2.7 Statistical analysis

All experimental data are presented as the means ± SEM. Statistical analysis of the data was performed with the two‐tailed independent Student's *t* test or ANOVA analysis using GraphPad Prism 5.0 (GraphPad Software). A value of *P* < .05 was considered to indicate a statistically significant difference.

## 3 RESULTS

### 3.1 Identification of pulmonary macrophages in the injured lung tissue

**Figure 1:**
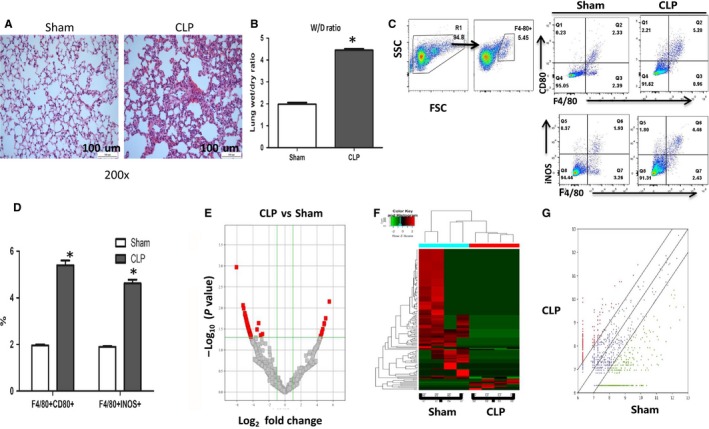
CircRNA expression profile of pulmonary macrophages in Sham versus CLP mice. A, Lungs were fixed, sectioned and stained with H&E. Representative sections are shown from sham and CLP mice (Original magnification, × 200). B, Evaluation of wet/dry weight ratio is compared between sham and CLP mice. All data are expressed as mean ± SEM (N = 8, *P < .05 vs Sham group). C, Macrophages in the lung homogenates were isolated and phenotyped by FACS analysis. Representative dot plots are shown for expression of percentage INOS and CD80 and their expressions on the F4/80 + macrophages in the lung homogenates. D, The percentage of F4/80 + INOS +and F4/80 + CD80 +macrophages were compared between the sham and CLP mice. All data are expressed as mean ± SEM (N = 4, *P < .05 vs Sham group). E, Volcano plot showing the differentially expressed circRNAs in the two groups [Plot of circRNA expression log2‐transformed fold‐changes (*x*‐axis) vs ‐log10 *P*‐value (*y*‐axis)]. The red dots represent the circRNAs having fold‐changes >2.0 and *P*‐values < .05 between the two groups of macrophages. F, Hierarchical cluster analysis revealed the expression profile of the dysregulated circRNAs in the two groups. G, The scatter plot shows the circRNA expression variation between the two groups. The values of *X* and *Y* axes in the scatter plot are the averaged normalized signal values of groups of samples (log2 scaled). The green lines are fold change lines. The circRNAs above the top green line and below the bottom green line indicated more than 1.5‐fold change of circRNAs between the two groups of samples

The ALI animal model was induced by CLP, and we observed that the CLP mice exhibited an increased ALI parameters as indicated by development of overt tissue oedema, infiltration of neutrophils (Figure 1A) and high lung wet/dry (W/D) ratio (Figure 1B) in comparison with SCLP animals. FACS analysis showed that the ratio of isolated pulmonary F4/80^+^ macrophages was 94.8% (Figure 1C). We found that there was a significantly increased ratio of M1 markers [INOS (inducible nitric oxide synthase) and CD80] in lung homogenates of CLP mice in comparison with sham mice (Figure 1D). Moreover, we observed a slightly increased ratio of M2 markers (CD206, Arg1 and FIZZ‐1) at mRNA and protein levels but there was no significant difference in lung macrophages between CLP mice and sham‐operated mice (data not shown).

### 3.2 Identification of differentially expressed circRNAs in pulmonary macrophages after ALI

A total of 4318 circRNAs were identified in mouse pulmonary macrophages at baseline. Of them, 1923 circRNAs were already included in the circBase or had been identified in previous studies, while 2395 circRNAs were first identified in the present study. Differential gene expression analysis revealed that expression levels of 137 circRNAs were significantly different, with 11 of them being up‐regulated and 126 down‐regulated (Table S1). The expression ratios (log2 scale) of those circRNAs in pulmonary macrophages are shown as volcano plots at different *P*‐values and fold‐change (Figure 1E), heat maps (Figure 1F) and scatter plots (Figure 1G).

### 3.3 CircRNAs gene symbols and pathway analysis

GO enrichment analysis showed that the up‐regulated circRNAs were involved in multiple biological functions such as regulation of mitochondrion distribution and Notch binding (Figure S1A), while the down‐regulated circRNAs were mainly involved in the biological processes, such as histone H3K27 methylation and H3K24 trimethylation (Figure S1B). On the other hand, KEGG analysis revealed that TGF‐beta signalling pathway was related to the up‐regulated circRNAs (Figure S2A), while sphingolipids and NOD‐like receptor signalling pathways were observed in the down‐regulated circRNAs (Figure S2B).

### 3.4 qRT‐PCR validation of the differentially expressed circRNAs

**Figure 2:**
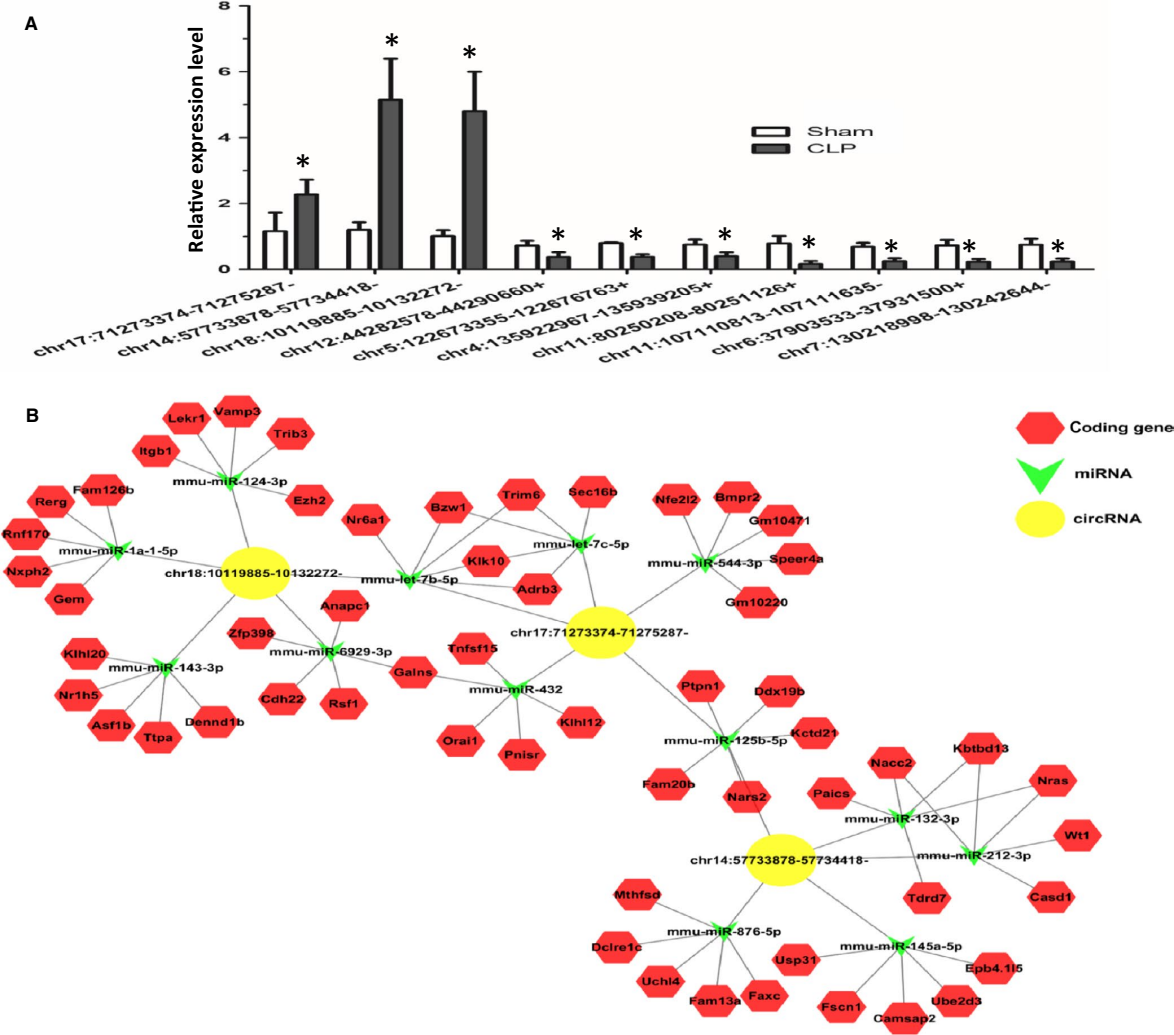
Validation of circRNA expression by RT‐PCR and network of circular RNAs and the predicted binding miRNAs. A, Changes in CircRNA expression were confirmed using RT‐PCR for select circRNAs in the Sham and CLP groups. Bars represent mean ± SEM (n = 3), *P < .05. B, The three up‐regulated circRNAs were annotated in detail according to the circRNA/miRNA interaction information by Cytoscape. Based on the miRNA prediction and bioinformatics analyses, we show the top 5 miRNAs may be regulated by three up‐regulated circRNAs and top 5 target genes of each miRNA, respectively

The top 10 most differentially expressed circRNAs including three up‐regulated circRNAs and seven down‐regulated circRNAs (Table S2) were further confirmed by qRT‐PCR; their expression levels were consistent with the sequencing results (Figure 2A).

### 3.5 Identification of circRNA‐targeting miRNAs and construction of circRNA‐miRNA‐mRNA networks

All circRNAs identified in the present study were found to have miRNA binding sites (data not shown). The top five miRNA binding sites for dysregulated circRNAs were predicted (data not shown). Table S3 lists the seven validated down‐regulated circRNAs and their top five miRNA binding sites.

To better explore and predict the potential function of the three most significant up‐regulated circRNAs in CLP‐induced ALI model, a tree diagram of circRNAs and their potential binding miRNAs is generated in Figure 2B. In the network map, we showed the top five miRNAs that potentially bind to the circRNA and five most likely targeted genes to each miRNA (Figure 2B).

## 4 DISCUSSION

The present study aimed to identify the expression patterns of circRNAs in the pulmonary macrophages of mice with CLP‐induced lung injury. In total, 11 significantly upregulated and 126 significantly down‐regulated circRNAs were identified. Of those circRNAs, 10 differentially expressed circRNAs were selected for further validation using qRT‐PCR and all of them were confirmed to be significantly different. All of the differentially expressed circRNAs were identified to contain MREs to sponge different miRNAs. Of them, the three best candidate circRNAs were selected and their circRNA‐miRNA‐mRNA networks were constructed using bioinformatics tools. The up‐regulated chr17:71273374‐71275287‐, chr14:57733878‐57734418‐ and chr18:10119885‐10132272‐ were predicted to negatively regulate the function of mmu‐miR‐7c‐5p, mmu‐miR‐132‐3p and mmu‐miR‐124‐3p separately. All of these miRNAs have all been identified thus far to promote anti‐inflammatory responses and M2 polarization.^8^, ^9^, ^10^, ^11^ Given that three up‐regulated circRNAs have the potential to inhibit the function of M2 polarization‐related miRNAs, it can be hypothesized that down‐regulation of these targeted miRNAs can promote M1 polarization and aggregate the lung injury.

Besides, among the enriched GO terms found in this study, the biological process as histone H3K27 methylation and H3K24 trimethylation was identified. Recent studies have revealed that histone methylation changes in monocytes alter chromatin structure and ultimately direct the expression of numerous transcription factors crucial in the differentiation and polarization process, thus regulation of inflammatory responses.^12^ On this basis, it is proposed that the circRNAs identified in this study may contribute to the epigenetic control of macrophage polarization and activation in sepsis‐induced lung injury and inflammation. Moreover, our KEGG analysis indicated that the typical TGF‐β signalling pathway, which is reported to be involved in the promotion of alveolar macrophage development and homeostasis,^13^ is also related to the up‐regulated circRNAs. This suggests that TGF‐β signalling pathway may be able to regulate lung macrophages behaviours through alternation of circRNAs expression. This hypothesis is worthy of further investigations.

In conclusion, the present study is the first to profile circRNA expression patterns in macrophage activation and contributes to the understanding of the role of circRNAs in macrophage polarization under septic lung injury. Our findings suggest that circRNAs may play a key role in the pathological process of sepsis‐induced lung injury.

## AUTHOR CONTRIBUTION

LT, JB and XB conceived and designed the study; QZ, ZL and HS involved in analysis and interpretation; and XH, SZ, NL, QS and LT drafted the manuscript for important intellectual content.

## Data Availability

I confirm that my article contains a Data Availability Statement even if no data is available (list of sample statements) unless my article type does not require one (eg, Editorials, Corrections, Book Reviews, etc.).
