# Supplementary material for: Characteristics of circular RNA expression of pulmonary macrophages in mice with sepsis‐induced acute lung injury
Source: J Cell Mol Med. 2019 Aug 14;23(10):7111–5. doi: 10.1111/jcmm.14577 (PMC6787439; doi:10.1111/jcmm.14577)
Supplement: Supplementary file 1 [file JCMM-23-7111-s001.docx]

**
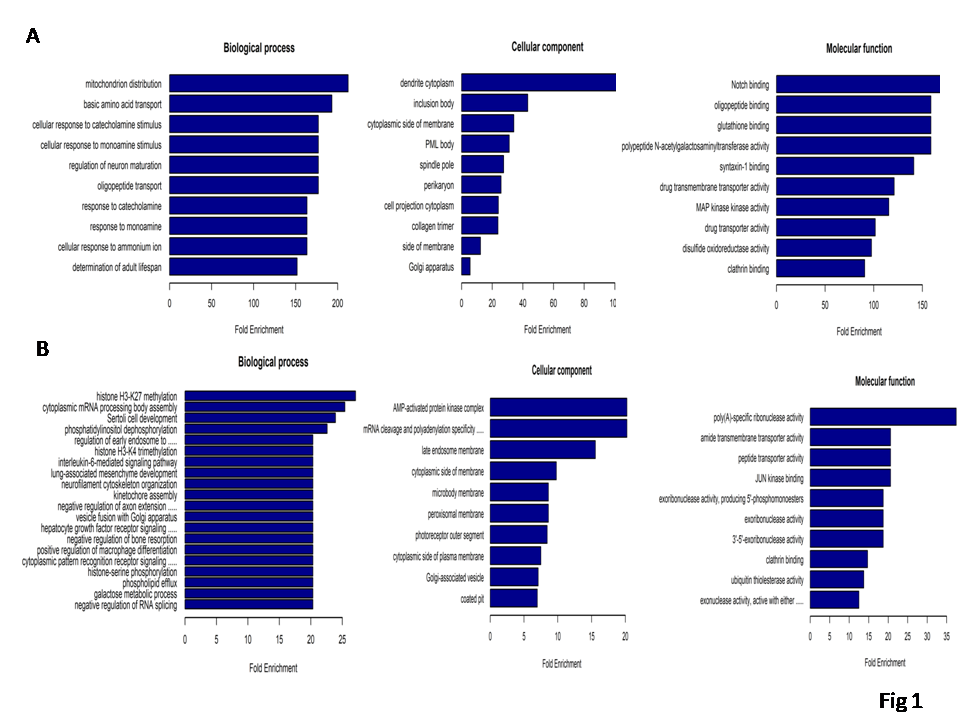
**

**Fig.1.GO enrichment analysis for dysregulated circRNAs gene symbols.** (A) Most significantly enriched GO [–log10 (P value)] terms of upregulated circRNAs gene symbols according to molecular function, cellular component and biological process. (B) Most significantly enriched GO [–log10 (P value)] terms of downregulated circRNAs gene symbols according to molecular function, cellular component and biological process.

**
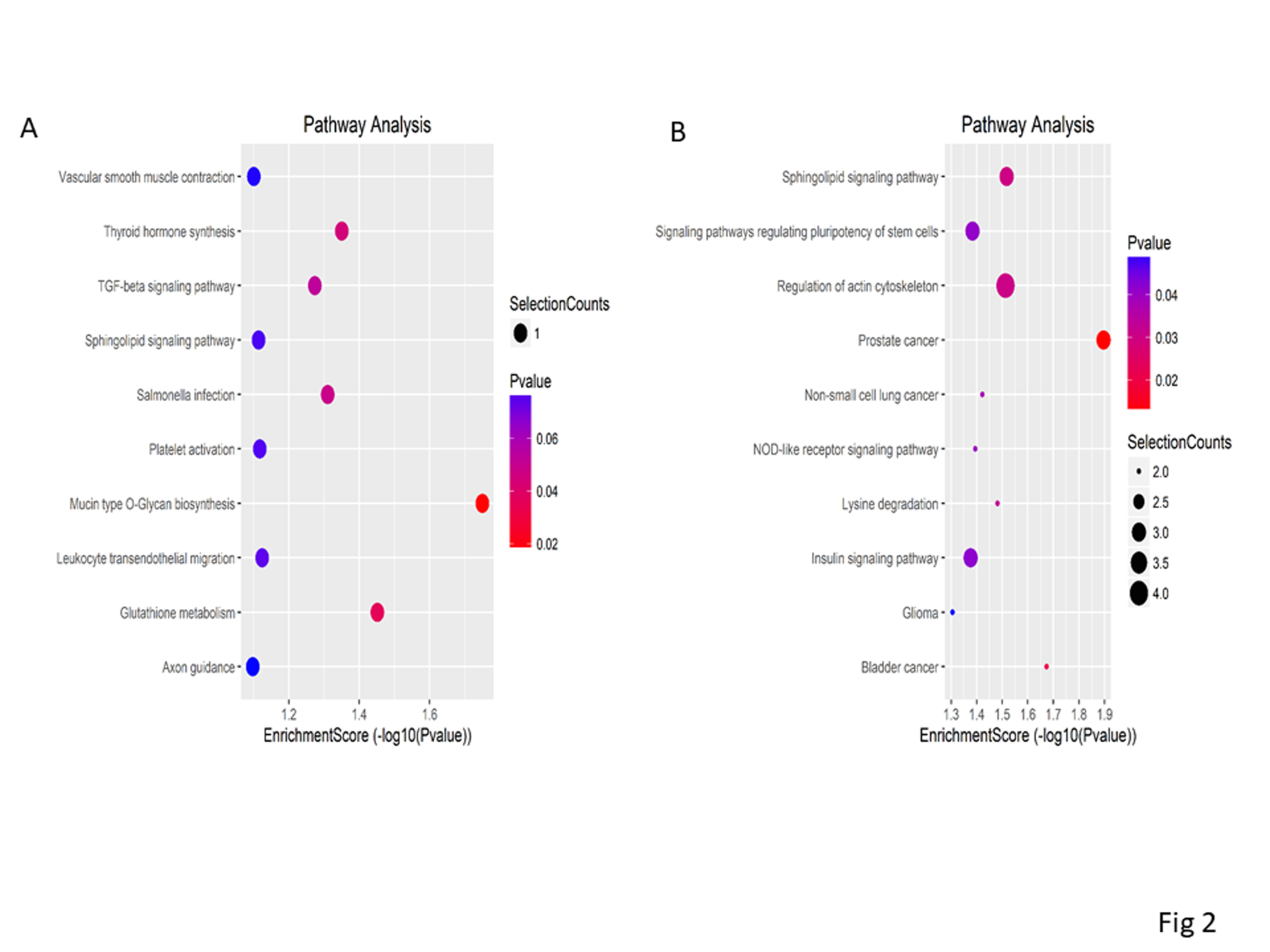
**

**Fig. 2. KEGG pathway analysis of the target genes of dysregulated circRNAs**. CircRNAs with 2‑fold change and P<0.05 were selected from the dysregulated circRNAs, and the target genes of these circRNAs were identified using bioinformatics tools. (A) The top 10 pathways of the target genes of upregulated circRNAs were identified using KEGG analysis according to the enrichment score**.** (B)The top 10 pathways of the target genes of downregulated circRNAs were identified using KEGG analysis according to the enrichment score**.**
