# Supplementary material for: Characteristics of circular RNA expression of pulmonary macrophages in mice with sepsis‐induced acute lung injury
Source: J Cell Mol Med. 2019 Aug 14;23(10):7111–5. doi: 10.1111/jcmm.14577 (PMC6787439; doi:10.1111/jcmm.14577)
Supplement: Supplementary file 2 [file JCMM-23-7111-s002.docx]

**Table 1 137 differentially expressed circRNAs in macrophages after ALI**

| **CircRNAID** | **LogFc** | **LogCPM** | **P value** | **Regulation** | **circRNA Type** | **Gnee Symbol** |
| --- | --- | --- | --- | --- | --- | --- |
| chr17:71273374-71275287- | 5.526794 | 10.58906 | 0.007061 | up | exonic | Emilin2 |
| chr18:10119885-10132272- | 5.073838 | 10.34004 | 0.017667 | up | exonic | Rock1 |
| chr14:74775581-74786390- | 4.909116 | 10.25009 | 0.023053 | up | exonic | Lrch1 |
| chr5:127601858-127609061- | 4.878213 | 10.23601 | 0.024434 | up | exonic | Slc15a4 |
| chr14:57733878-57734418- | 4.707192 | 10.15232 | 0.031949 | up | exonic | Lats2 |
| chr15:91705106-91712862+ | 4.609267 | 10.10812 | 0.036927 | up | exonic | Lrrk2 |
| chr8:86865721-86866760- | 4.570216 | 10.09733 | 0.040685 | up | intronic | N4bp1 |
| chr5:137325323-137325674- | 4.464232 | 10.04806 | 0.046192 | up | exonic | Slc12a9 |
| chr8:33669153-33689452+ | 4.433451 | 10.0341 | 0.047701 | up | exonic | Gsr |
| chr5:121407193-121409204+ | 4.417244 | 10.02683 | 0.048547 | up | exonic | Naa25 |
| chr5:25261935-25263484+ | 4.416071 | 10.02742 | 0.048997 | up | exonic | Galnt11 |
| **CircRNAID** | **LogFc** | **LogCPM** | **P value** | **Regulation** | **circRNA Type** | **Gnee Symbol** |
| chr7:103813439-103826793- | -6.06156 | 10.92392353 | 0.001077101 | down | sense overlapping | Hbb-bt |
| chr18:56807664-56812075- | -5.2403 | 10.41159432 | 0.008657313 | down | exonic | March3 |
| chr5:150466244-150471610+ | -5.15875 | 10.36668485 | 0.010232328 | down | exonic | Fry |
| chr4:135922967-135939205+ | -5.02603 | 10.29680816 | 0.013307996 | down | sense overlapping | Fuca1 |
| chr14:73262529-73269198- | -5.01878 | 10.29189316 | 0.013419346 | down | exonic | Rb1 |
| chr6:38440455-38502408+ | -5.01374 | 10.2920115 | 0.013729683 | down | sense overlapping | Ubn2 |
| chr3:121228310-121235764- | -5.00778 | 10.2906245 | 0.014007403 | down | exonic | Tmem56 |
| chr12:86256868-86269061+ | -4.99328 | 10.28317187 | 0.014403012 | down | exonic | Gpatch2l |
| chr14:78982236-78984302+ | -4.94023 | 10.25238724 | 0.015616393 | down | exonic | Vwa8 |
| chr11:80250208-80251126+ | -4.91294 | 10.24004538 | 0.016545132 | down | exonic | Rhot1 |
| chr14:62925130-62934402+ | -4.89229 | 10.2290917 | 0.01712575 | down | exonic | Wdfy2 |
| chr12:54923040-54935581- | -4.84054 | 10.20527187 | 0.018987144 | down | exonic | Baz1a |
| chr4:99748686-99753191+ | -4.83971 | 10.20299929 | 0.018813097 | down | exonic | Alg6 |
| chr11:107110813-107111635- | -4.83383 | 10.20199589 | 0.019217641 | down | exonic | Bptf |
| chr1:80634200-80648332- | -4.83041 | 10.20114894 | 0.019429452 | down | exonic | Dock10 |
| chr12:44282578-44290660+ | -4.79235 | 10.1818957 | 0.020687696 | down | exonic | Pnpla8 |
| chr19:8696618-8697350- | -4.78658 | 10.18004083 | 0.021016044 | down | intergenic |  |
| chr8:90932249-90933862+ | -4.7724 | 10.1740686 | 0.021660814 | down | exonic | Chd9 |
| chr11:94067893-94069035+ | -4.77154 | 10.17147919 | 0.021396796 | down | exonic | Spag9 |
| chr7:98243949-98276937- | -4.77154 | 10.17147919 | 0.021396796 | down | sense overlapping | Acer3 |
| chr9:108541688-108542586+ | -4.76434 | 10.16883 | 0.021773032 | down | exonic | Qrich1 |
| chr10:14004201-14076962+ | -4.7246 | 10.15306178 | 0.023740411 | down | sense overlapping | Hivep2 |
| chr8:25640649-25649703+ | -4.7246 | 10.15306178 | 0.023740411 | down | sense overlapping | Whsc1l1 |
| chr11:23320661-23349343+ | -4.72095 | 10.15135267 | 0.023879736 | down | exonic | Usp34 |
| chr1:160700712-160724189- | -4.71735 | 10.14668999 | 0.023513806 | down | exonic | Rabgap1l |
| chr4:6415077-6415568+ | -4.69968 | 10.13902649 | 0.024278783 | down | antisense | Nsmaf |
| chr14:72652016-72683771- | -4.68898 | 10.13575854 | 0.024992038 | down | exonic | Fndc3a |
| chr18:79086551-79087180- | -4.66891 | 10.126418 | 0.025783438 | down | exonic | Setbp1 |
| chr15:44501438-44511695+ | -4.66093 | 10.12139488 | 0.025851407 | down | exonic | Pkhd1l1 |
| chr7:100088064-100093484- | -4.65838 | 10.11933903 | 0.025785735 | down | exonic | Pold3 |
| chr18:10574875-10586041- | -4.6555 | 10.11991022 | 0.026258598 | down | exonic | Esco1 |
| chr14:49033271-49042549- | -4.63219 | 10.11069689 | 0.02753121 | down | exonic | Exoc5 |
| chr15:5160603-5177205+ | -4.62114 | 10.10357408 | 0.027563314 | down | exonic | Prkaa1 |
| chr17:26445394-26445590+ | -4.60103 | 10.09434042 | 0.028390873 | down | sense overlapping | Neurl1b |
| chr7:19853572-19853898- | -4.58026 | 10.08556067 | 0.029430283 | down | exonic | Ceacam16 |
| chr5:36543177-36572037- | -4.56596 | 10.0799026 | 0.030246366 | down | exonic | Tbc1d14 |
| chr12:71074936-71076069+ | -4.56195 | 10.07814185 | 0.030437526 | down | exonic | Arid4a |
| chr11:83365647-83369782+ | -4.53822 | 10.06735119 | 0.031470274 | down | exonic | Ap2b1 |
| chrX:47920161-47936488+ | -4.53822 | 10.06735119 | 0.031470274 | down | exonic | Ocrl |
| chr10:67186019-67186838+ | -4.53822 | 10.06735119 | 0.031470274 | down | intronic | Jmjd1c |
| chr7:110052711-110054116+ | -4.53822 | 10.06735119 | 0.031470274 | down | exonic | Ipo7 |
| chr11:54705126-54706018- | -4.53822 | 10.06735119 | 0.031470274 | down | intergenic |  |
| chr16:13629583-13647299- | -4.53822 | 10.06735119 | 0.031470274 | down | exonic | Parn |
| chr12:86256868-86267183+ | -4.53822 | 10.06735119 | 0.031470274 | down | exonic | Gpatch2l |
| chr2:154515817-154524099+ | -4.53822 | 10.06735119 | 0.031470274 | down | exonic | Cbfa2t2 |
| chr7:51887912-51943791+ | -4.53594 | 10.06552957 | 0.031365672 | down | exonic | Gas2 |
| chr8:95365314-95366422+ | -4.50929 | 10.05753504 | 0.033681197 | down | exonic | Mmp15 |
| chr5:122673355-122676763+ | -4.49643 | 10.04965423 | 0.033576112 | down | exonic | P2rx7 |
| chr14:54663587-54667161- | -4.495 | 10.0489421 | 0.033617925 | down | exonic | Acin1 |
| chr6:22339273-22343319- | -4.495 | 10.0489421 | 0.033617925 | down | exonic | Fam3c |
| chr7:45383804-45394815- | -4.495 | 10.0489421 | 0.033617925 | down | exonic | Snrnp70 |
| chr6:24616256-24666557- | -4.495 | 10.0489421 | 0.033617925 | down | sense overlapping | Wasl |
| chrY:90793079-90793680+ | -4.48891 | 10.04888845 | 0.034698295 | down | sense overlapping | Erdr1 |
| chr17:50047297-50055627- | -4.48464 | 10.04707432 | 0.0349087 | down | exonic | Rftn1 |
| chr7:132701347-132701621- | -4.47396 | 10.04051354 | 0.034786885 | down | intronic | Fgfr2 |
| chr8:111485090-111486995- | -4.47396 | 10.04051354 | 0.034786885 | down | exonic | Wdr59 |
| chr4:35083770-35084389- | -4.47396 | 10.04051354 | 0.034786885 | down | exonic | Mob3b |
| chr18:75114790-75126919+ | -4.47177 | 10.04162212 | 0.035550493 | down | exonic | Dym |
| chr5:135440022-135451635- | -4.46746 | 10.03980145 | 0.035768013 | down | exonic | Hip1 |
| chr6:37903533-37931500+ | -4.45541 | 10.0334148 | 0.035937241 | down | exonic | Trim24 |
| chr16:10991150-10991479- | -4.4525 | 10.03199201 | 0.036023193 | down | intronic | Litaf |
| chr14:103262702-103291362- | -4.45057 | 10.03032979 | 0.035838965 | down | exonic | Mycbp2 |
| chr11:116847997-116848623- | -4.42877 | 10.02175137 | 0.037122961 | down | antisense | Mettl23 |
| chr2:167963628-167967827+ | -4.42877 | 10.02175137 | 0.037122961 | down | exonic | Ptpn1 |
| chr2:126917211-126917881- | -4.42877 | 10.02175137 | 0.037122961 | down | exonic | Sppl2a |
| chr13:59512324-59544451- | -4.42422 | 10.02067751 | 0.037635641 | down | exonic | Agtpbp1 |
| chr18:67519291-67530484- | -4.41391 | 10.01631577 | 0.038151881 | down | exonic | Spire1 |
| chr5:96091584-96098313- | -4.38826 | 10.00641646 | 0.039785602 | down | exonic | Cnot6l |
| chr5:74545412-74557149+ | -4.38217 | 10.00277797 | 0.039684676 | down | exonic | Fip1l1 |
| chr12:73318977-73352599+ | -4.38217 | 10.00277797 | 0.039684676 | down | sense overlapping | Slc38a6 |
| chr19:3914066-3980974+ | -4.38217 | 10.00277797 | 0.039684676 | down | sense overlapping | Unc93b1 |
| chr10:83304304-83316789- | -4.38217 | 10.00277797 | 0.039684676 | down | exonic | Slc41a2 |
| chr17:74433735-74441986- | -4.38217 | 10.00277797 | 0.039684676 | down | exonic | Nlrc4 |
| chr19:6341903-6343176+ | -4.37725 | 10.00158263 | 0.040256612 | down | exonic | Map4k2 |
| chr14:32973536-32986748- | -4.36059 | 9.994643122 | 0.04113406 | down | exonic | Wdfy4 |
| chr13:103835560-103836938- | -4.3591 | 9.99394519 | 0.041183638 | down | intronic | Erbb2ip |
| chr18:38299754-38309713+ | -4.3591 | 9.99394519 | 0.041183638 | down | exonic | Rnf14 |
| chr4:155157370-155160956- | -4.3591 | 9.99394519 | 0.041183638 | down | intronic |  |
| chr10:40434304-40436212+ | -4.3591 | 9.99394519 | 0.041183638 | down | exonic | Cdk19 |
| chr11:4517741-4531305- | -4.34003 | 9.987102479 | 0.04262593 | down | exonic | Mtmr3 |
| chr17:74585953-74599967+ | -4.33406 | 9.983589455 | 0.042501004 | down | exonic | Birc6 |
| chr1:91300528-91308815+ | -4.33406 | 9.983589455 | 0.042501004 | down | exonic | Scly |
| chr6:38461475-38491931+ | -4.33406 | 9.983589455 | 0.042501004 | down | exonic | Ubn2 |
| chr1:58397676-58401511+ | -4.3247 | 9.982350469 | 0.044168342 | down | exonic | Bzw1 |
| chr7:34344784-34345112- | -4.3247 | 9.982350469 | 0.044168342 | down | sense overlapping | Lsm14a |
| chrM:6293-6464+ | -4.3247 | 9.982350469 | 0.044168342 | down | sense overlapping | DQ539915 |
| chr11:61903907-61909946- | -4.31635 | 9.977151059 | 0.043814255 | down | exonic | Akap10 |
| chr11:23351592-23355098+ | -4.31161 | 9.975284723 | 0.04410377 | down | exonic | Usp34 |
| chr17:80144980-80150170+ | -4.3104 | 9.976681509 | 0.045058157 | down | exonic | Galm |
| chr1:39555478-39571942- | -4.31011 | 9.974594094 | 0.044157257 | down | sense overlapping | Rnf149 |
| chr16:14391318-14396424+ | -4.31011 | 9.974594094 | 0.044157257 | down | sense overlapping | Abcc1 |
| chr4:72157463-72170770- | -4.31011 | 9.974594094 | 0.044157257 | down | exonic | Tle1 |
| chr9:80092267-80130858+ | -4.31011 | 9.974594094 | 0.044157257 | down | exonic | Senp6 |
| chr11:21263199-21278060+ | -4.30561 | 9.974788371 | 0.045360231 | down | exonic | Vps54 |
| chr11:70917315-70925912+ | -4.3008 | 9.972893488 | 0.045665093 | down | exonic | Rabep1 |
| chr2:172444647-172447113+ | -4.29828 | 9.970634231 | 0.045202507 | down | exonic | Rtfdc1 |
| chr9:70703287-70722771+ | -4.29598 | 9.970996859 | 0.045972778 | down | exonic | Adam10 |
| chr12:59050268-59051708- | -4.29598 | 9.970996859 | 0.045972778 | down | exonic | Trappc6b |
| chr3:109562743-109578364+ | -4.29598 | 9.970996859 | 0.045972778 | down | exonic | Vav3 |
| chr1:170156924-170166948- | -4.28867 | 9.966871253 | 0.045810633 | down | exonic | Uap1 |
| chr10:25479009-25489093+ | -4.28433 | 9.964181852 | 0.045605605 | down | exonic | Epb4.1l2 |
| chr10:51491910-51492298+ | -4.28433 | 9.964181852 | 0.045605605 | down | sense overlapping | Lilrb4 |
| chr7:130218998-130242644- | -4.26263 | 9.958049868 | 0.04818145 | down | exonic | Fgfr2 |
| chr9:72591751-72593360+ | -4.26263 | 9.958049868 | 0.04818145 | down | exonic | Rfx7 |
| chr10:81108748-81118229+ | -4.25943 | 9.955019051 | 0.047440179 | down | exonic | Map2k2 |
| chr13:112487709-112490642 | -4.25943 | 9.955019051 | 0.047440179 | down | exonic | Il6st |
| chr1:30947111-30947735- | -4.25943 | 9.955019051 | 0.047440179 | down | intronic | Ptp4a1 |
| chr2:24806214-24838868- | -4.25943 | 9.955019051 | 0.047440179 | down | exonic | Ehmt1 |
| chr3:119740315-119753116- | -4.25943 | 9.955019051 | 0.047440179 | down | exonic | Ptbp2 |
| chr5:111182924-111183431+ | -4.25943 | 9.955019051 | 0.047440179 | down | exonic | Ttc28 |
| chr6:22318934-22343319- | -4.25943 | 9.955019051 | 0.047440179 | down | exonic | Fam3c |
| chr14:37131538-37133803- | -4.25943 | 9.955019051 | 0.047440179 | down | exonic | Ghitm |
| chr3:29590677-29591243+ | -4.25943 | 9.955019051 | 0.047440179 | down | intronic | Egfem1 |
| chr2:4519724-4520368+ | -4.25943 | 9.955019051 | 0.047440179 | down | intronic | Frmd4a |
| chr15:50822105-50831958- | -4.25943 | 9.955019051 | 0.047440179 | down | exonic | Trps1 |
| chr11:103683792-103687633- | -4.25943 | 9.955019051 | 0.047440179 | down | exonic | Gosr2 |
| chr10:50748806-50754122+ | -4.25769 | 9.956138687 | 0.048512754 | down | exonic | Ascc3 |
| chr6:3545510-3578915+ | -4.25273 | 9.954225742 | 0.048847201 | down | exonic | Ccdc132 |
| chr9:107691130-107691448- | -4.23553 | 9.946426317 | 0.049347417 | down | intronic | Sema3f |
| chr2:14236732-14237530+ | -4.23398 | 9.945745132 | 0.049408314 | down | intronic | Mrc1 |
| chr4:111746141-111747803+ | -4.23398 | 9.945745132 | 0.049408314 | down | exonic | Spata6 |
| chr2:14257004-14266535+ | -4.23398 | 9.945745132 | 0.049408314 | down | exonic | Mrc1 |
| chr5:73194986-73221072- | -3.49564 | 10.9247333 | 0.032527335 | down | exonic | Fryl |
| chr16:93849130-93853898+ | -3.29262 | 11.30234997 | 0.02296384 | down | exonic | Morc3 |
| chr2:128776286-128779872+ | -3.04469 | 10.98612383 | 0.044731459 | down | exonic | Mertk |
| chr9:66540062-66566903- | -2.80905 | 11.41497175 | 0.041753104 | down | exonic | Usp3 |
