# Supplementary material for: Characteristics of circular RNA expression of pulmonary macrophages in mice with sepsis‐induced acute lung injury
Source: J Cell Mol Med. 2019 Aug 14;23(10):7111–5. doi: 10.1111/jcmm.14577 (PMC6787439; doi:10.1111/jcmm.14577)
Supplement: Supplementary file 3 [file JCMM-23-7111-s003.docx]

**Table 2. Primer sequences of the 10 circRNAs used in real-time PCR are listed in the 5'-3' direction**

| Name | 5‘-’Forward | 3’-Reverse |
| --- | --- | --- |
| chr17:71273374-71275287- | ACGCTCAAATCGCACTCC | CGACCTTCTCCTCCAGCA |
| chr14:57733878-57734418- | GCCAACGAGTCAGGCACT | CATGCTTTCAGAACAACTTCC |
| chr18:10119885-10132272- | TCAGTGGGCTTGGGAAAC | TGGGATTTTAATACTTCG |
| chr4:135922967-135939205+ | GGTGGAGTTTGCGTGGAC | GCACAGCTGCTCCCAACT |
| chr11:80250208-80251126+ | GGCAGAAGAAAATTCGTGATG | TCCAGTTTTCCCACAGCC |
| chr11:107110813-107111635- | GGTGCCTGGTGTGACTGA | GGAAATTCAAGGGGTGGG |
| chr12:44282578-44290660+ | TCCGAATCCTCACCATTGA | TCATTCCATTCCTGAATGCTT |
| chr5:122673355-122676763+ | CATCATGGAACCCAAGCC | CAGTGGCCAAGCCAAAGT |
| chr6:37903533-37931500+ | ACAGCAGGAAGTGGCTGG | TTGACTGCAAACTGGGCA |
| chr7:130218998-130242644- | TGGAAAGTGTGGTCCCGT | CGTACGCTTCTGGTTGGG |
| GAPDH | GGCCTCCAAGGAGTAAGACC | AGGGGAGATTCAGTGTGGTG |
