# Supplementary material for: Characteristics of circular RNA expression of pulmonary macrophages in mice with sepsis‐induced acute lung injury
Source: J Cell Mol Med. 2019 Aug 14;23(10):7111–5. doi: 10.1111/jcmm.14577 (PMC6787439; doi:10.1111/jcmm.14577)
Supplement: Supplementary file 4 [file JCMM-23-7111-s004.docx]

**Table 3. Top five miRNA binding sites for seven validated downregulated circRNAs**

| CircRNA | MRE1 | MRE2 | MRE3 | MRE4 | MRE5 |
| --- | --- | --- | --- | --- | --- |
| chr4:135922967-135939205+ | mmu-miR-5124b | mmu-miR-6407 | mmu-miR-6413 | mmu-miR-24-3p | mmu-miR-7216-5p |
| chr11:80250208-80251126+ | mmu-miR-145a-5p | mmu-miR-145b | mmu-miR-467a-3p | mmu-miR-669f-3p | mmu-miR-669b-3p |
| chr11:107110813-107111635- | mmu-miR-6978-3p | mmu-miR-877-3p | mmu-miR-485-3p | mmu-miR-181d-5p | mmu-miR-7664-5p |
| chr12:44282578-44290660+ | mmu-miR-6932-3p | mmu-miR-26a-2-3p | mmu-miR-22-5p | mmu-miR-26b-3p | mmu-miR-7039-3p |
| chr5:122673355-122676763+ | mmu-miR-1197-3p | mmu-miR-6539 | mmu-miR-5627-3p | mmu-miR-27a-3p | mmu-miR-27b-3p |
| chr6:37903533-37931500+ | mmu-miR-1903 | mmu-miR-7650-3p | mmu-miR-6946-3p | mmu-miR-465d-5p | mmu-miR-223-3p |
| chr7:130218998-130242644- | mmu-miR-680 | mmu-miR-6933-5p | mmu-miR-7036a-3p | mmu-miR-20b-3p | mmu-miR-106a-3p |
| circRNA, circular RNA; MRE, miRNA binding site; miR, microRNA. | | | | | |
